# *Ceratobasidium* sp. GS2 exudates elicit downstream transcriptional and physiological responses in seeds of *Gymnadenia conopsea* (*Orchidaceae*)

**DOI:** 10.3897/imafungus.17.191381

**Published:** 2026-07-17

**Authors:** Yaoyao Wang, Xin Qian, Jiaxin Li, Haoran Wang, Aiyiwei Yang, Luna Yang, Jiaxin Liu, Xiaoke Xing

**Affiliations:** 1 State Key Laboratory for Quality Ensurance and Sustainable Use of Dao-di Herbs, Institute of Medicinal Plant Development, Chinese Academy of Medical Sciences & Peking Union Medical College, Beijing, China State Key Laboratory for Quality Ensurance and Sustainable Use of Dao-di Herbs, Institute of Medicinal Plant Development, Chinese Academy of Medical Sciences & Peking Union Medical College Beijing China

**Keywords:** Common symbiosis signaling pathway, fungal exudates, orchid mycorrhiza, symbiotic germination

## Abstract

*Orchidaceae* is a highly valuable horticultural and medicinal plant family worldwide; however, large-scale propagation and conservation remain severely limited. Orchid seeds depend on symbiotic fungi for germination, and pre-symbiotic communication is essential for establishing a successful association, a process that remains poorly understood. In this study, exudates were collected from the germination-promoting fungus *Ceratobasidium* sp. GS2 and applied to seeds of the terrestrial orchid *Gymnadenia conopsea* to investigate downstream responses. Multi-omics approaches, including RNA-seq, metabolomics, and phylogenetic analysis, combined with biological validation, revealed that the exudates elicited transcriptional and physiological responses in *G. conopsea* seeds, potentially promoting dormancy release. Exposure to fungal exudates increased the levels of brassinosteroids, cytokinins, and fatty acids in seeds. Exogenous hormone application confirmed that brassinosteroids and cytokinins promote fungal colonization and facilitate symbiotic seed germination. Phylogenetic analysis revealed the conservation of symbiotic genes in partially mycoheterotrophic orchids, and functional characterization confirmed the role of GcRAM2 in *G. conopsea*. These findings provide new insights into the mechanisms underlying orchid mycorrhizal symbiosis.

## Introduction

*Orchidaceae* Juss., encompassing over 31,000 species (Freudenstein 2025; Govaerts 2026), evolved approximately 85 million years ago and developed the unique trait of endosperm-free seeds (Pérez-Escobar et al. 2024). In nature, orchid seeds typically rely on mycorrhizal fungi for successful germination and development (Zhao et al. 2024), a strategy known as mycoheterotrophy (Leake and Cameron 2010). In some green orchids, photosynthetically fixed carbon can be transferred from the plant to its fungal partner (Cameron et al. 2007; Li et al. 2022), whereas achlorophyllous orchids remain entirely dependent on carbon obtained from fungi (Merckx 2012). Compared with ectomycorrhiza (*EcM*) and arbuscular mycorrhizal (*AM*) associations, orchid mycorrhiza (*OrM*) interactions are less well studied, likely due to the unique life cycle of orchids and their diverse trophic strategies (Valadares et al. 2021). In recent years, substantial progress has been made in elucidating the evolutionary origin of orchids and the nature of nutrient exchange, both of which are crucial for their conservation and the maintenance of plant diversity (Wei et al. 2025); however, the mechanisms underlying the formation of this symbiosis are poorly understood.

Orchid seed germination represents a unique developmental process closely linked to mycorrhizal symbiosis, as these minute seeds lack endosperm and contain immature embryos incapable of synthesizing sufficient nutrients on their own (Yeung et al. 2018). Symbiotic fungi (e.g., *Ceratobasidium* spp. and *Tulasnella* spp.) colonize the embryo through the seed micropyle and may further penetrate internal tissues via rhizoids arising from the protocorm (Li et al. 2020; Gao et al. 2023). After colonization, hyphae proliferate within the embryo’s cortical cells in the lower-middle region of the protocorm, forming highly coiled pelotons (Fochi et al. 2017). Protocorm formation is a hallmark of orchid seed germination, and successful establishment with a compatible mycorrhizal symbiont promotes subsequent seedling development (Ma et al. 2022). Specifically, the protocorm functions as a dual-purpose hub integrating symbiosis and meristematic activity: the apical region is responsible for meristem establishment and the initiation of root and tuber primordia, while the basal region specializes in symbiotic fungal colonization (Gao et al. 2023). Fungi colonizing the protocorm base extend into newly formed root cortical cells through vascular bundle gaps or cortical interstices, ultimately forming new pelotons in the root mid-cortex and exhibiting the same preference for parenchyma-cell colonization as observed in the protocorm base (Li et al. 2020; Gao et al. 2023). This structural division of labor closely parallels that of *AM* roots, in which meristems continuously generate new cortical cells to sustain fungal colonization (Gutjahr et al. 2008; Pumplin et al. 2010), highlighting the evolutionary conservation of the structural basis for intracellular symbiosis.

Pre-symbiotic communication influences plant–microbe interactions. The common symbiosis signaling pathway (CSSP) serves as a universal signaling hub for intracellular symbioses, regulating plant interactions with intracellular symbionts (e.g., *AM*, *OrM*, and rhizobia) (Radhakrishnan et al. 2020). In classical mutualisms, such as *AM* and rhizobium–legume symbioses, fungal or bacterial exudates trigger the conserved CSSP in host plants, resulting in transcriptomic changes and morphological modifications essential for colonization (Kosuta et al. 2003; Oláh et al. 2005; Gutjahr et al. 2009). Specifically, germinating spore exudates (GSEs) from *AM* fungi activate MAPK signaling, induce calcium spiking, and trigger the expression of symbiotic genes (e.g., DMI1/2/3), thereby facilitating root development and nutrient uptake (Kosuta et al. 2003; Oláh et al. 2005; Sun et al. 2015; Díaz et al. 2025). Similarly, rhizobial exudates promote root hair curling and the formation of infection threads through the CSSP while modulating root growth via phytohormones such as auxin and cytokinin (Singh et al. 2021; Wang et al. 2022). These symbiotic signals in exudates were termed mycorrhizal factors and later identified as lipo-chitooligosaccharides (LCOs) or chito-oligosaccharides (COs) (Maillet et al. 2011; Zhang et al. 2025).

**Figure 1.**
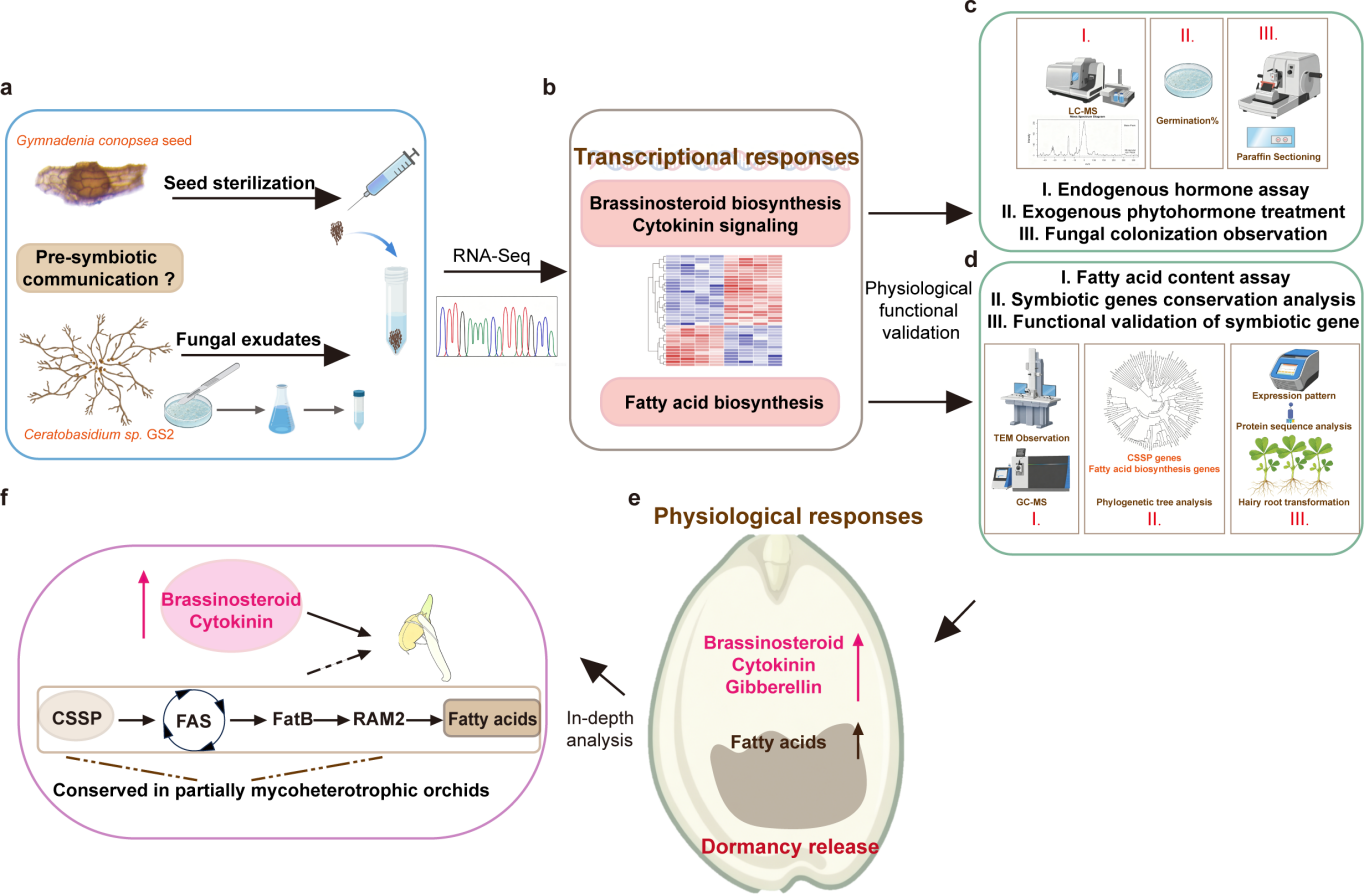
Schematic diagram of the study. **a** Schematic of the experimental model (*Gymnadenia conopsea* seeds and *Ceratobasidium* sp. GS2) and treatment setup. Sterilized *G. conopsea* seeds were treated with concentrated fungal exudates from GS2, with sterile water as the control. Samples were prepared for RNA-seq analysis (*n* = 4), transmission electron microscopy (TEM) observation (*n* = 3), endogenous phytohormone assay (*n* = 5), and fatty acid content assay (*n* = 5) **b** Core transcriptional responses induced by fungal exudates. Transcriptome analysis revealed significant upregulation of genes involved in brassinosteroid (BR) biosynthesis, cytokinin (CK) signaling, and fatty acid biosynthesis pathways **c** Physiological validation of phytohormone functions. I. Endogenous phytohormone quantification via LC-MS/MS (*n* = 5); II. exogenous phytohormone treatment and symbiotic germination rate assay (*n* = 12 plates per treatment); III. fungal colonization observation using paraffin sectioning and toluidine blue staining (*n* = 10 protocorms per treatment) **d** Functional validation of fatty acid metabolism and symbiotic genes. I. Fatty acid content determination via GC-MS (*n* = 5) and lipid droplet observation via TEM; II. conservation analysis of symbiotic genes through phylogenetic tree construction; III. functional validation of the symbiotic gene GcRAM2 via *Medicago truncatula* hairy root transformation (*n* = 6–8 independent experiments) **e** Summary of physiological responses in *G. conopsea* seeds. Exposure to fungal exudates significantly increased the levels of brassinosteroids, cytokinins, gibberellins, and fatty acids, potentially promoting seed dormancy release **f** Potential pathways for *G. conopsea* symbiotic germination. BR and CK positively regulate the symbiotic germination of *G. conopsea* seeds. Partially mycoheterotrophic orchids such as *G. conopsea* retain the AM-derived CSSP and functional GcRAM2 for fatty acid biosynthesis, which likely play important roles in this process.

The mechanism of pre-symbiotic communication between orchid seeds and symbiotic fungi in *OrM* associations remains largely unexplored. In this study, the aim was to investigate how orchid seeds respond to exudates from germination-promoting fungi in the absence of physical contact. To answer this question, the germination-promoting fungus *Ceratobasidium* sp. GS2 and seeds of the terrestrial orchid *Gymnadenia conopsea* were used as an interaction model. Mycelial exudates were collected for further analysis. The data confirm that *OrM* fungal exudates elicit downstream transcriptional and physiological responses in *G. conopsea* seeds, potentially facilitating seed dormancy release, and that exposure to exudates increases fatty acid levels in seeds, presumably via the CSSP (Fig. 1). These findings provide novel insights and enhance understanding of the pre-symbiotic communication mechanisms underlying *OrM* symbiosis, while offering partial theoretical support for further research on *OrM* interactions.

## Materials and methods

### Fungal material and culture

The fungus *Ceratobasidium* sp. GS2, isolated from the root of *Gymnadenia conopsea* (Gao et al. 2020), was used in this study. It has been deposited at the Mycological Herbarium of the Institute of Microbiology, Chinese Academy of Sciences (CGMCC 16089), with NCBI GenBank accession number OK655751.1. The other tested strains included five *Ceratobasidium* strains, GZ15, GZ16 (isolated from *G. conopsea*), MF13756, RJXL42, and LL71 (isolated from *Dendrobium chrysotoxum*, *Anoectochilus roxburghii*, and *Anoectochilus lylei*, respectively), and one *Tulasnella* strain, GB32 (isolated from *G. conopsea*) (Gao et al. 2020; Zhao et al. 2025b). The ITS region of nuclear ribosomal DNA was amplified and sequenced using the primer pair ITS1-OF and ITS4-OF (Taylor and McCormick 2008). Fungal ITS sequences are provided in Suppl. material 2: table S1. A maximum-likelihood tree was constructed in MEGA v. 12.0.0 (Suppl. material 1: fig. S1).

All fungi were cultured on potato dextrose agar (PDA) in 9 cm Petri dishes at 23 °C in the dark prior to inoculation. The PDA medium contained 200 g L^-1^ potato, 20 g L^-1^ glucose (Yuanye, Shanghai, China), and 15 g L^-1^ agar (Biotopped, Beijing, China). For symbiotic germination of *G. conopsea* seeds, seeds were evenly spread on one side of the symbiotic culture medium. Small pieces of fungal inoculum were placed at the center of the Petri dish, which was then sealed and incubated at 23 °C in the dark on oatmeal agar (OMA; 5 g L^-1^ rolled oats, 12 g L^-1^ agar (Biotopped, Beijing, China)) medium.

### Seed collection

Mature seeds of *G. conopsea* were collected in mid-August 2025 from the Baihua Mountain Nature Reserve, Beijing, China. The taxonomic identity of *G. conopsea* populations at this site has been previously documented (Xing et al. 2019).

Ten healthy, mature plants were randomly selected, and 10 intact, unopened seed capsules were harvested from each individual. Following harvest, capsules were surface-sterilized by immersion in 3% (v/v) sodium hypochlorite solution for 5 min and then rinsed 5–6 times with sterile distilled water. After sterilization, capsules were air-dried at room temperature and stored in 5 mL centrifuge tubes with desiccant to maintain low humidity. Seeds were stored in the dark at 4 °C until use.

### Preparation of fungal exudates and seed treatment

Fungal hyphal exudates were used for untargeted metabolomics, phytohormone and lipid content analyses, root hair branching assays, RNA-seq, and transmission electron microscopy (TEM) experiments. The method for collecting exudates was adapted from previous literature with minor modifications (Cope et al. 2019). Initially, fungal cultures were grown on OMA medium, with one plug of similar size per 90 mm Petri dish. After 7 days, when the mycelium had fully covered the Petri dish, 100 mg of mycelium from each dish was transferred into 100 mL of sterile water in a sterile glass bottle as a biological replicate. All liquid cultures were incubated at 23 °C in the dark for 10 days without shaking. The hyphal exudates in the sterile water were filtered, collected, freeze-dried, and then resuspended in sterile water and concentrated 60-fold. They were subsequently sterilized using a 0.22 μm syringe filter and stored at –20 °C for future use.

To investigate the effect of exudates on seeds, seeds were placed in a syringe fitted with a sieve at the distal tip to prevent seed loss, then disinfected with 1% NaClO for 2–3 min and rinsed 5–6 times with sterile water. For each biological replicate of seed treatment, three independent 100 mL aliquots of the original hyphal exudates were pooled and concentrated to a final volume of 5 mL. A total of four biological replicates were set up for the experiment. Each biological replicate in the treatment group received 5 mL of concentrated exudates, while the control replicates received 5 mL of sterile water, followed by incubation for 18 days. After treatment, seeds from each biological replicate (100 mg) were washed five times with sterile water, blotted to remove excess moisture, frozen in liquid nitrogen, and used for RNA-seq and hormone analysis.

### Root hair branching assays

Because legume root hair branching assays have been widely validated for LCO detection in both rhizobial and *AM* systems (Maillet et al. 2011; Cope et al. 2019; Rush et al. 2020), and no standardized equivalent assay currently exists for orchids, *Medicago truncatula* was chosen as a sensitive and reproducible model to assess canonical LCO bioactivity in *OrM* fungal exudates.

To detect potential mycorrhizal factors (LCOs) in *OrM* fungi, a root hair branching assay was conducted, and the frequency of root hair branching was subsequently quantified. One milliliter of sterile exudate was applied to the roots of 1-week-old *M. truncatula* seedlings. Treated plants were incubated at room temperature for 24 h, with the plates partially covered with aluminum foil to keep the roots in the dark while allowing the leaves to be exposed to light. After incubation, the basal 3 cm of roots from each treatment were examined. Sterile water was used as a negative control.

### Untargeted metabolomics analysis of GS2 exudates

To analyze the composition of GS2 exudates, an untargeted metabolomics approach was employed. Four biological replicates were included in the analysis. For each biological replicate, a 1 mL exudate sample was freeze-dried, concentrated, and treated with a precipitant to remove proteins. The samples were resuspended in prechilled 80% methanol (Thermo Fisher, Germany), vortexed, incubated on ice for 5 min, and centrifuged at 15,000 × *g* and 4 °C for 20 min. The supernatant was diluted to 53% methanol using LC-MS-grade water, transferred to fresh tubes, and centrifuged again. Finally, the supernatant was injected into the LC-MS/MS system for metabolomic analysis. The ultra-high-performance liquid chromatography (UHPLC) system (Vanquish, Thermo Fisher, Germany) was coupled to an Orbitrap Q Exactive HF mass spectrometer (Thermo Fisher, Germany) at Novogene Co., Ltd. (Beijing, China), and metabolites were analyzed in both positive and negative ionization modes using linear gradients and appropriate solvents. Data were processed using XCMS for peak alignment and quantification, and metabolites were annotated with the KEGG (Kanehisa et al. 2016), HMDB (Wishart et al. 2022), and LIPID Maps (Conroy et al. 2024) databases. The results of the untargeted metabolomics analysis of GS2 exudates are presented in Suppl. material 2: table S2.

### Construction and *de novo* assembly of RNA-seq data for exudates-treated seeds

To characterize the transcriptome of seeds treated with GS2 exudates, RNA-seq analysis was conducted. The cDNA libraries were sequenced using the DNBSEQ-T7 (MGI Tech Co., Ltd., Shenzhen, China) platform. *De novo* transcriptome assembly was performed with Trinity, and assembly quality was evaluated using BUSCO (Benchmarking Universal Single-Copy Orthologs). Gene functional annotation was performed using seven major databases: Nr, Nt, Pfam, KOG/COG, Swiss-Prot, KEGG, and GO (Harris et al. 2004; Boutet et al. 2007; Kanehisa et al. 2016; Mistry et al. 2021; Sayers et al. 2022; Galperin et al. 2025). Gene expression levels were quantified as fragments per kilobase of transcript per million mapped fragments (FPKM) values. Differential expression analysis was performed using DESeq2 v. 1.6.1 in R v. 3.1.3 (Love et al. 2014) by Novogene Co., Ltd. (Beijing, China), using thresholds of |log_2_(fold change)| ≥ 1 and padj ≤ 0.05. KEGG functional enrichment analysis of the differentially expressed genes (DEGs) was conducted using KOBAS software (Mao et al. 2005), and pathways were considered enriched when the *p* value was ≤ 0.1 (Suppl. material 2: table S3).

**Figure 2.**
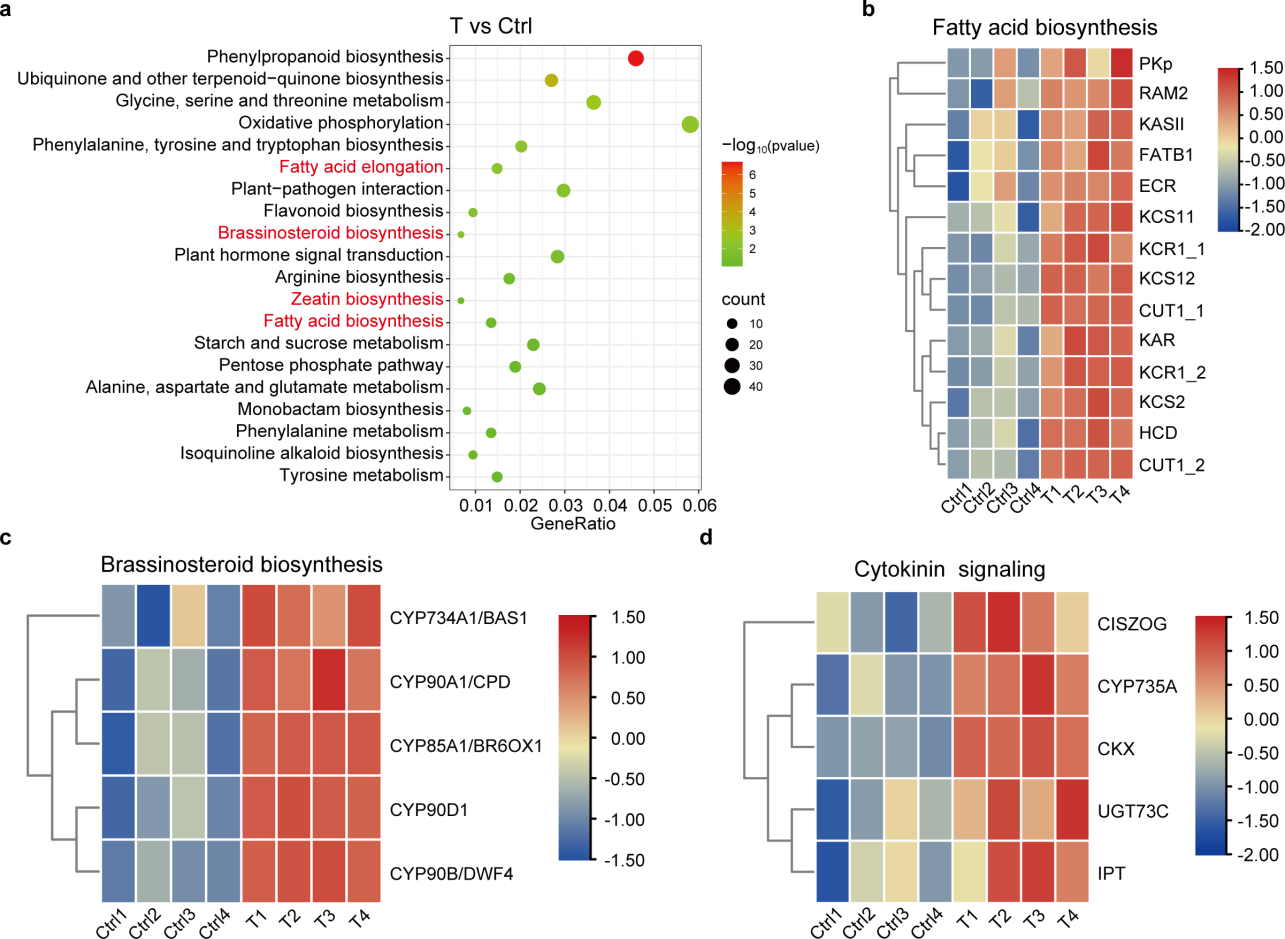
Transcriptome analysis of *G. conopsea* seeds in response to fungal exudate exposure. **a** KEGG pathway enrichment analysis of upregulated genes in *G. conopsea* seeds in response to GS2 exudates. Ctrl, control group; T, treated group **b** Heatmap showing the expression patterns of fatty acid biosynthesis-related genes in Ctrl and treated samples **c, d** Heatmaps showing the expression patterns of genes involved in brassinosteroid biosynthesis (**c**) and cytokinin signaling (**d**) pathways in Ctrl and treated samples based on KEGG pathway analysis. FPKM values were normalized for the heatmap using a red–yellow–blue color gradient (red = high expression, blue = low expression), with row clustering, gene/sample labels, and a color bar.

DEGs were mapped to KEGG pathways for enrichment analysis; significantly enriched pathways were identified, and all associated genes were extracted to construct target pathway gene sets. Finally, heatmaps were generated to visualize the expression patterns of these genes across samples. Expression patterns of candidate genes were visualized using heatmaps generated and analyzed with TBtools v. 2.483 (Chen et al. 2023). Visualization parameters for the heatmaps were as follows: FPKM values were log_2_-transformed (with an offset of 1), followed by row-wise normalization. Row clustering dendrograms, together with gene and sample labels, were retained for clarity. A red–yellow–blue color gradient was applied, with red representing relatively high expression and blue representing relatively low expression, and a labeled color bar was included to indicate the numerical range of relative expression values. Gene IDs and annotation information for Fig. 2b–d are provided in Suppl. material 2: table S4.

### Time-series transcriptomic analysis during symbiotic germination of *G. conopsea* seeds

**Figure 3.**
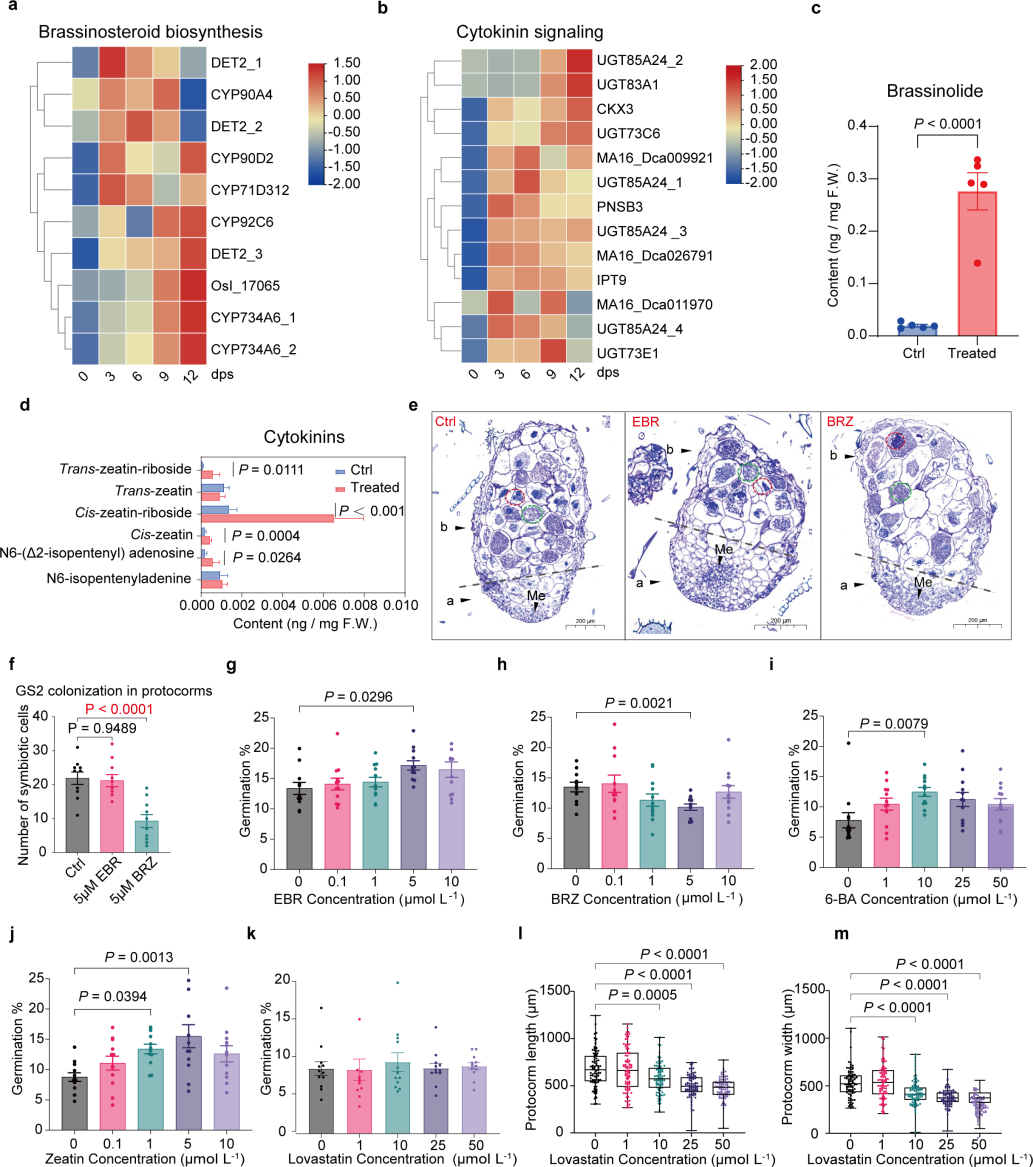
Brassinosteroid and cytokinin are associated with *G. conopsea* symbiotic seed germination. **a, b** Heatmaps showing the expression patterns of genes related to BR biosynthesis (**a**) and CK signaling (**b**) in symbiotic germination samples collected at 0, 3, 6, 9, and 12 dps. FPKM values (averaged across three biological replicates) were normalized for the heatmap, which used a red–yellow–blue color gradient (red = high expression, blue = low expression), with row clustering, gene/sample labels, and a color bar **c, d** Profiles of endogenous BR (**c**) and bioactive CKs (**d**) in the control and exudate exposure groups of *G. conopsea* seeds. Bars represent mean ± SEM (*n* = 5). Two-tailed two-sample *t*-test. Significance was defined as *p* < 0.05. F.W., fresh weight **e** Longitudinal sections of toluidine blue-stained symbiotic protocorms of *G. conopsea* treated with EBR (5 μmol L^-1^) and BRZ (5 μmol L^-1^) at 30 dps, showing active pelotons (green circles) and senescent pelotons (red circles). “a” indicates the chalazal end, and “b” indicates the micropylar end **f** Quantification of symbiotic cells colonized by strain GS2 under control, 5 μmol L^-1^ EBR, and 5 μmol L^-1^ BRZ treatments at 30 dps. Bars represent mean ± SEM (*n* = 10) **g–k** Effects of exogenous treatments with EBR, BRZ, 6-BA, zeatin, and lovastatin on the *in vitro* germination rate of *G. conopsea* seeds at 21 dps. Bars represent mean ± SEM (*n* = 12 plates) **l, m** Statistical analysis of protocorm length and width of *G. conopsea* under exogenous lovastatin treatment at 30 dps. Bars represent mean ± SEM (*n* = 90–100 protocorms). Statistical significance was determined using one-way ANOVA followed by Tukey’s test. Significance was defined as *p* < 0.05 (**f–m**). Scale bars: 200 μm (**e**).

**Figure 4.**
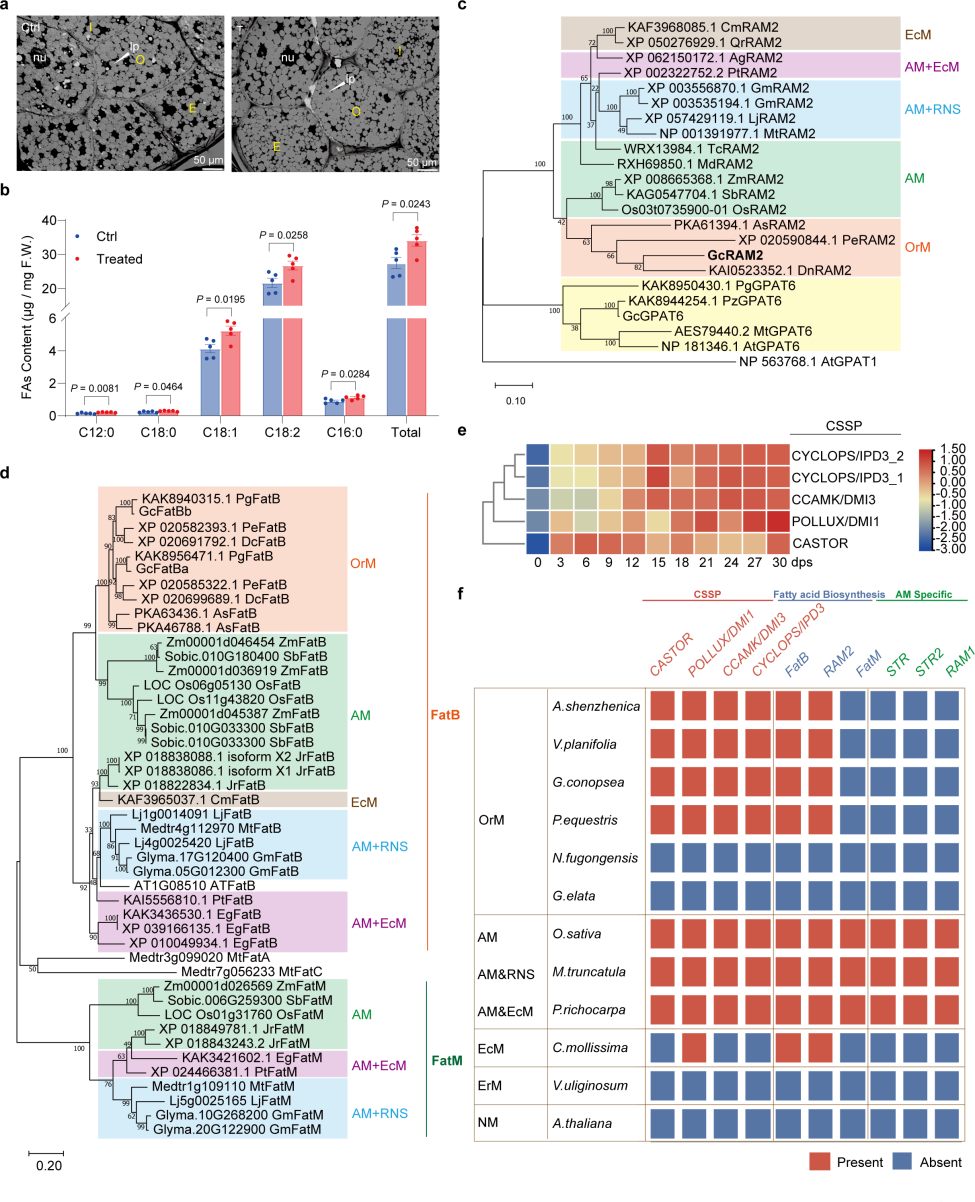
Fungal exudate exposure increased fatty acid levels in *G. conopsea* seeds, likely via the CSSP. **a**TEM images showing the cellular structures and contents of seeds in Ctrl (left) and treated (right) samples. I, inner cortex cell; O, outer cortex cell; E, epidermal cell; nu, nucleus; lp, lipid droplet **b** Fatty acid profile in seeds treated with fungal exudates and in control seeds. Bars represent mean ± SEM (*n* = 5). Two-tailed two-sample *t*-test. Significance was defined as *p* < 0.05. F.W., fresh weight **c** Evolutionary analysis of glycerol-3-phosphate acyltransferase (*RAM2*) across species with *AM*, *EcM*, *OrM*, *AM*+*EcM*, and *AM*+*RNS* symbioses, inferred using the maximum-likelihood method. GPAT, glycerol-3-phosphate acyltransferase **d** Evolutionary analysis of fatty acid thioesterase M (*FatM*) and acyl-ACP thioesterase B (*FatB*) across species with *AM*, *EcM*, *OrM*, *AM*+*EcM*, and *AM*+*RNS* symbioses, inferred using the maximum-likelihood method **e** Heatmap of CSSP genes (*CASTOR*, *POLLUX/DMI1*, *CCaMK/DMI3*, and *CYCLOPS/IPD3*) showing their expression patterns in symbiotic germination samples at 0, 3, 6, 9, 12, 15, 18, 21, 24, 27, and 30 dps. FPKM values (averaged across three biological replicates) were normalized for the heatmap, which used a red–yellow–blue color gradient (red = high expression, blue = low expression), with row clustering, gene/sample labels, and a color bar. *CCaMK*, Ca^2+^- and CaM-dependent serine/threonine protein kinase; *CASTOR* and *POLLUX/DMI1*, ion channel proteins; *CYCLOPS/IPD3*, DNA-binding transcriptional activator **f** Schematic representation of the conservation of symbiotic genes among different types of symbioses. *AM*, arbuscular mycorrhizal; *EcM*, ectomycorrhiza; *RNS*, root-nodule symbiosis; *OrM*, orchid mycorrhiza; *ErM*, ericoid mycorrhiza; *NM*, mutualism abandonment. Scale bar: 50 μm (**a**).

Time-series transcriptomic raw data from the symbiotic germination of *G. conopsea* seeds (used in this study, Figs 3a, b, 4e) were retrieved from the NCBI Sequence Read Archive (SRA) under accession number PRJNA1218826, and the *de novo*-assembled seed transcriptome data generated with Trinity were retrieved from the GenBank Transcriptome Shotgun Assembly (TSA) database under accession number GLDP00000000. These transcriptomic datasets were originally generated and reported in a previous study (Zhao et al. 2025a) and are reanalyzed here for the present research. The acquisition of target genes is described in the preceding section. Gene IDs and annotation information for the transcriptome data are provided in Suppl. material 2: tables S4, S5.

### Quantification of endogenous phytohormones

To assess endogenous phytohormone levels in *G. conopsea* seeds treated with GS2 exudates, targeted metabolomics analysis was conducted to quantify phytohormones. Each biological replicate in the treatment group received 5 mL of exudates, while each replicate in the control group received 5 mL of sterile water, with five biological replicates per group. Samples (100 mg) were ground in a 2 mL grinding tube, and 498 μL of 80% methanol, along with 2 μL of internal standard (2 μg/mL), was added. The mixture was vortexed, sonicated for 1 h at low temperature, and centrifuged. The supernatant (100 μL) was transferred to a vial and diluted with 60 μL of water.

LC-MS/MS analysis was performed using an AB SCIEX QTRAP 6500+ system (AB SCIEX, USA) coupled to a Waters BEH C18 column (2.1 × 100 mm, 1.7 μm, Waters, USA). The mobile phases consisted of 0.1% formic acid in water (A) and acetonitrile (B) (Thermo Fisher Scientific, Waltham, MA, USA), with a flow rate of 0.35 mL/min and the following gradient: 70% A for 0 min, 40% A for 3 min, and 0% A for 10 min. The mass spectrometer was operated in both positive and negative ionization modes, with an IonSpray voltage of ± 5,500 V and a curtain gas pressure of 35 psi. Data were analyzed with Sciex OS software (AB SCIEX, USA), and standard curves for each target hormone were constructed based on the peak area vs. concentration relationship. Detailed information on quantitative detection is provided in Suppl. material 2: table S6.

### Preparation and staining of paraffin sections

To examine the effect of exogenous hormone treatment on GS2 colonization, tissues were fixed in 4% paraformaldehyde, dehydrated, and embedded in paraffin. Embedded tissues were sectioned and treated with a series of xylene and ethanol solutions for clearing. Sections were stained with toluidine blue, slightly differentiated in 1% glacial acetic acid, and mounted with neutral gum. Microscopic examination and image analysis were conducted using a Nikon Eclipse TI-SR inverted fluorescence microscope (Nikon Corporation, Tokyo, Japan). Paraffin sectioning and staining followed Chieco et al. (1993) and Gao et al. (2023), with a few modifications for fungal colonization observation.

### Sample preparation for electron microscopy

Seed lipid distribution after exudate treatment was examined by fixing seeds in 5% glutaraldehyde solution containing 20% EDTA, followed by vacuum infiltration, according to Cao et al. (2021), with minor modifications. After fixation, the seeds were washed three times with phosphate buffer (PB) for 10 min, followed by fixation for an additional 2 h and the same washing procedure. Dehydration was carried out using a graded acetone series (50%, 70%, 90%, 100%, and 100%) for 10 min at each step. Infiltration with Spurr resin was performed using 50% resin for 3 h, 70% resin overnight, and 100% resin for 24 h, repeated twice (Spurr 1969). Embedding was completed, followed by resin polymerization. Ultrathin sections (70 nm) were cut, stained, and examined using an HT7700 transmission electron microscope (Hitachi, Tokyo, Japan).

### Exogenous phytohormone treatment

To investigate the effects of exogenous hormone treatment on *G. conopsea* seed symbiotic germination, 24-epibrassinolide (Solarbio, Beijing, China), zeatin (Yeasen, Shanghai, China), 6-BA (Biotopped, Beijing, China), and the hormone synthesis inhibitors brassinazole (Yuanye, Shanghai, China) and lovastatin (MedChemExpress, Monmouth Junction, NJ, USA) were applied. All compounds were dissolved in DMSO and stored at –20 °C at a concentration of 100 mM. Stock solutions were prepared at serial concentrations to ensure that the same volume was added for treatments with different hormone concentrations. For each hormone treatment, an equivalent concentration of DMSO was used as the 0 mM control. The concentrations and treatment protocols for exogenous phytohormones and inhibitors were adapted from previous studies (Zhang et al. 2014; Groszyk and Szechyńska-Hebda 2021; Azzam et al. 2022; Zhang et al. 2022; Ying et al. 2024; Yousaf et al. 2024), with minor adjustments based on preliminary experimental results.

For symbiotic germination of *G. conopsea* seeds, germination was defined as seed swelling and testa rupture (Stewart and Zettler 2002). Germination rate was calculated as the number of germinated seeds divided by the total number of seeds, with seeds sown on a single plate per treatment. Symbiotic cells were quantified following Yamamoto et al. (2017). Briefly, from stained paraffin sections of 30 days post-symbiosis (dps) protocorms, two uniformly sized protocorms were randomly selected per treatment, with five biological replicates.

### Fatty acid content assay

The analytical method followed Yan et al. (2022) and Zhu et al. (2022), with the following steps: fatty acid content and composition in *G. conopsea* seeds were analyzed by extracting 50 mg of tissue with dichloromethane/methanol 1:1 (v/v) (dichloromethane, Sinopharm, Shanghai, China; methanol, Fisher Chemical, Waltham, MA, USA). After sonication, centrifugation, and nitrogen evaporation, the extract was methylated with sodium hydroxide (Sinopharm, Shanghai, China) in methanol, then mixed with *n*-hexane (Fisher Chemical, Waltham, MA, USA) and analyzed using GC-MS (Agilent 8890-7000D, Agilent Technologies, Santa Clara, CA, USA). Fatty acid identification was achieved by matching acquired mass spectra against the NIST 17 mass spectral database, while quantification was performed using MassHunter software via external calibration curves in a targeted analysis of 51 fatty acid methyl esters (ANPEL Laboratory Technologies Inc., Shanghai, China).

### Phylogenetic tree construction

Protein sequences and reference fungal internal transcribed spacer (ITS) sequences were retrieved from the NCBI database (https://www.ncbi.nlm.nih.gov) (Sayers et al. 2025) and aligned using ClustalW (Thompson et al. 1994). Maximum-likelihood (ML) phylogenetic trees were constructed for both datasets using MEGA v. 12.0.0 (Kumar et al. 2024). Neighbor-joining (NJ) (Saitou and Nei 1987) and maximum-parsimony (MP) trees were used as initial trees for heuristic searches, and bootstrap analysis was conducted with 1,000 replicates to assess the robustness of tree topologies (Felsenstein 1985).

For protein sequence analysis, the Jones-Taylor-Thornton (JTT) model (Jones et al. 1992) of amino acid substitution was employed, and evolutionary distances were calculated using the same JTT model. For fungal ITS sequence analysis, the Tamura-Nei model (Tamura and Nei 1993) of nucleotide substitution was used, and evolutionary distances were computed using the Kimura 2-parameter (K2P) model (Kimura 1980).

### Gene expression analysis

To investigate the tissue-specific expression of *GcRAM2* in *G. conopsea*, quantitative real-time PCR (qRT-PCR) was conducted to determine its relative expression levels. Total RNA was extracted using TRIzol reagent (CWBIO, Beijing, China), and first-strand cDNA synthesis was performed with 1 μg of RNA using ReverTra Ace qPCR RT Master Mix with gDNA Remover (TOYOBO, Osaka, Japan). qRT-PCR was conducted on a MyiQ real-time PCR system (Bio-Rad, Hercules, CA, USA) using 2 × UltraSYBR Mixture (CWBIO, Beijing, China). Gene expression was normalized to the reference gene Actin 4 (ID: TRINITY_DN11909_c0_g1) in *G. conopsea*. Primer sequences are listed in Suppl. material 2: table S7.

### *M. truncatula* hairy root transformation

The *M. truncatula* ram2 mutant used in this study has been described previously (Wang et al. 2012). The wild-type background of the ram2 mutant is Jemalong A17. *M. truncatula* chimeric transgenic plants were generated by hairy root transformation as described by Boisson-Dernier et al. (2001). *M. truncatula* seeds were treated with H_2_SO_4_ for 8–12 min, rinsed, and then treated with sodium hypochlorite diluted 1:10 for 3 min, followed by washing with sterile water. Seeds were placed on 1% water agar at 4 °C for 3 days, then transferred to a 22 °C incubator overnight to allow root growth to approximately 1 cm before transformation. The constructs were individually introduced into *Agrobacterium rhizogenes* strain Arqua-1. After 3–4 weeks, composite plants were transferred to a greenhouse (22 °C with a 16 h light/8 h dark photoperiod), grown in a 1:1 perlite–sand mixture, and inoculated with approximately 400 *Rhizophagus irregularis* spores per plant. *AM* colonization was analyzed after 60 days using trypan blue staining for infection rate quantification (Phillips and Hayman 1970).

*R. irregularis* was propagated using a carrot hairy root culture system, according to the protocol described by Bécard and Fortin (1988). Cultures were maintained on MSR medium at 26 °C in continuous darkness (Declerck et al. 1996). The spore-bearing medium was suspended in sodium citrate–citric acid buffer (pH 6.0, Sinopharm, Shanghai, China) at a buffer-to-medium volume ratio of 2:1 to 5:1. After agitation until fully suspended, the mixture was filtered, the hyphae were disrupted, and the spores were subsequently harvested.

### Plasmid construction

For overexpression of *GcRAM2* and *MtRAM2* in the *M. truncatula* ram2 mutant, cDNA sequences of *GcRAM2* and *MtRAM2* were amplified by PCR using the primers listed in Suppl. material 2: table S7. The PCR products were cloned into the pENTR/SD/D-Topo vector (Invitrogen, Waltham, MA, USA). These two fragments were then introduced into pK7WG2R plasmids containing the *LjUBQ* promoter (Karimi et al. 2002; Wang et al. 2025) via Gateway LR reactions (Invitrogen, Waltham, MA, USA).

### Statistical analysis

All statistical analyses were performed using GraphPad Prism v. 10.1.2 software (Boston, MA, USA; https://www.graphpad.com). Data are presented as mean ± SEM, with statistically significant *p* values marked above each bar. For comparisons between two groups, unpaired two-tailed Student’s *t*-tests were applied. For comparisons among multiple groups, one-way analysis of variance (ANOVA) was conducted, followed by Tukey’s test for post hoc pairwise comparisons to determine statistical significance. A *p* value < 0.05 was considered statistically significant. Detailed statistical information corresponding to each analysis is provided in the corresponding figure legends. Data were confirmed to follow a normal distribution, and no significant differences in variance were observed between groups for individual comparisons.

## Results

### Transcriptional responses of *Gymnadenia conopsea* seeds to fungal exudate exposure

Transcriptome data showed that 18,051 genes were downregulated, 5,172 genes were upregulated, and 65,627 genes showed no significant change when samples with prolonged exposure to concentrated GS2 exudates (T) were compared with control (Ctrl) samples (Suppl. material 1: fig. S2a). RNA-seq analysis revealed that upregulated genes were predominantly enriched in starch and sucrose metabolism, fatty acid biosynthesis, fatty acid elongation, plant hormone signal transduction, brassinosteroid (BR) biosynthesis, and zeatin biosynthesis (Fig. 2a). GO analysis of differentially expressed genes revealed enrichment in catalytic, oxidoreductase, transferase, isomerase, lyase, and hydrolase activities, as well as cell wall organization and carbohydrate metabolism processes (Suppl. material 1: fig. S2b), all of which are potentially involved in seed dormancy release.

As shown in the heatmap (Fig. 2b), genes related to fatty acid biosynthesis—such as *PKp*, *KASII*, *KAR*, *FatB*, and *RAM2*—as well as fatty acid elongation genes—such as *KCS/CUT*, *KCR*, *HCD*, and *ECR*—were upregulated by exposure to fungal exudates. In the BR biosynthesis pathway (Fig. 2c), key genes such as *CYP90B*/*DWF4*, *CYP90A1*/*CPD*, *CYP85A1*/*BR6OX1*, and *CYP90D1* were upregulated. In the cytokinin signaling pathway (Fig. 2d), *IPT* and *CYP735A* were significantly upregulated. Additionally, genes involved in CK metabolism and regulation, including *cisZOG*, *UGT73C*, and *CKX*, were also induced.

Root hair branching assays were used to detect putative mycorrhizal factors, specifically potential LCOs, in *OrM* fungal exudates. As shown in Suppl. material 1: fig. S3a, roots of *M. truncatula* responded to exudates from GS2, other *Ceratobasidium* strains (MF13756, GZ15, GZ16, RJXL42, and LL71), and the *Tulasnella* strain GB32 by inducing root hair branching. All of these exudates elicited root hair branching in *M. truncatula*.

Untargeted metabolomic profiling of exudates from the germination-promoting fungus GS2 identified diverse metabolite classes. Lipids and lipid-like molecules were the most abundant (979 metabolites), followed by organoheterocyclic compounds (673) and organic acids and derivatives (591). Within the lipid category, fatty acyls (39.63%), prenol lipids (35.34%), and steroids and steroid derivatives (16.65%) were predominant (Suppl. material 1: fig. S3b).

### Brassinosteroid and cytokinin are associated with symbiotic seed germination

A time-series transcriptome analysis of seed symbiotic germination with GS2 at 3, 6, 9, and 12 days post-symbiosis (dps) showed that brassinosteroid (BR) biosynthetic and cytokinin (CK) signaling pathways were significantly upregulated prior to fungal colonization, which began at 12 dps (Fig. 3a, b).

The levels of endogenous brassinolide and bioactive CKs in seeds treated with GS2 exudates were further assessed. Brassinolide content significantly increased, reaching 14 times that of the control group (Fig. 3c). Bioactive CKs, including *trans*-zeatin-riboside, *cis*-zeatin-riboside, *cis*-zeatin, and *N*^6^-(Δ^2^-isopentenyl)adenosine, were also elevated, with *cis*-zeatin-riboside showing the highest proportion and being 4.7 times higher in the treated group compared to the control (Fig. 3d). To investigate the effect of exogenous hormones on GS2 colonization, protocorm paraffin sections were stained with toluidine blue (Fig. 3e). The brassinolide synthesis inhibitor brassinazole (BRZ) treatment significantly reduced the number of colonized cells in protocorms (Fig. 3f), indicating a decrease in the colonization rate. Exogenous 24-epibrassinolide (EBR) at 5 µmol L^-1^ increased the seed germination rate by 3.0% (Fig. 3g), while BRZ reduced the germination rate by 4.7% (Fig. 3h). Exogenous cytokinin treatments also enhanced the germination rate: 6-benzylaminopurine (6-BA) at 10 µmol L^-1^ increased it by 5.7%, and zeatin at 1 and 5 µmol L^-1^ increased it by 4.6% and 6.7%, respectively (Fig. 3i, j). However, treatment with EBR, BRZ, zeatin, and 6-BA did not induce significant changes in protocorm length and width (Suppl. material 1: fig. S4a–h). The cytokinin synthesis inhibitor lovastatin did not significantly affect the germination rate (Fig. 3k) but markedly inhibited protocorm growth, leading to a concentration-dependent reduction in protocorm size. At 50 µmol L^-1^, the protocorm diameter was the smallest, with a 29.5% decrease in length and a 28.8% decrease in width (Fig. 3l, m).

### Fungal exudate exposure increased fatty acid levels in *G. conopsea* seeds

Transmission electron microscopy (TEM) revealed an increased area ratio of lipid droplets in treated seeds relative to controls (Fig. 4a). Further quantitative analysis of fatty acids confirmed that the fatty acid content was higher in seeds treated with fungal exudates compared to control seeds (Fig. 4b). The results revealed that exudates induce the expression of *RAM2* and *FatB* in the fatty acid biosynthesis pathway (Fig. 2b). Fatty acid accumulation was also observed before GS2 colonization (12 dps), and genes related to fatty acid synthesis (such as glycerol-3-phosphate acyltransferase *RAM2* and acyl carrier protein thioesterase *FatB*) were induced in symbiotic seeds in a previous study (Wang et al. 2026). Phylogenetic analysis showed that *RAM2* is retained in all lineages but is lost in ericoid mycorrhiza (ErM). Although *RAM2* is broadly conserved among *OrM* lineages, it is absent in the fully mycoheterotrophic orchids *Neottia fugongensis* and *Gastrodia elata* (Fig. 4c, f). *FatM*, which encodes an acyl carrier protein thioesterase, was identified as an *AM*-specific enzyme (Fig. 4d); by contrast, *FatB* appears to have been recruited and retained across all mycorrhizal types (Fig. 4d). Key CSSP genes—*CCaMK*, *CASTOR*, *POLLUX*/*DMI1*, and *CYCLOPS*/*IPD3*—were upregulated as early as day 3 of symbiosis and remained highly expressed after fungal colonization (>12 dps), based on time-series transcriptomic data from the symbiotic germination of *Gymnadenia conopsea* seeds (Fig. 4e).

More in-depth analysis revealed that CSSP genes are generally widespread but show losses in subsets of *OrM*, *EcM*, and *ErM.* Notably, the fully mycoheterotrophic orchids *N. fugongensis* and *G. elata* lack the CSSP genes entirely. The fatty acid biosynthesis genes *FatB* and *RAM2* are shared by *AM* and *AM*-mixed symbioses but are absent from *ErM* and fully mycoheterotrophic orchids. In contrast, *FatM*, *RAM1*, *STR*, and *STR2* are *AM*-specific and are not detected in hosts of other symbiosis types (Fig. 4f).

### *GcRAM2* is functionally maintained in the orchid *G. conopsea*

**Figure 5.**
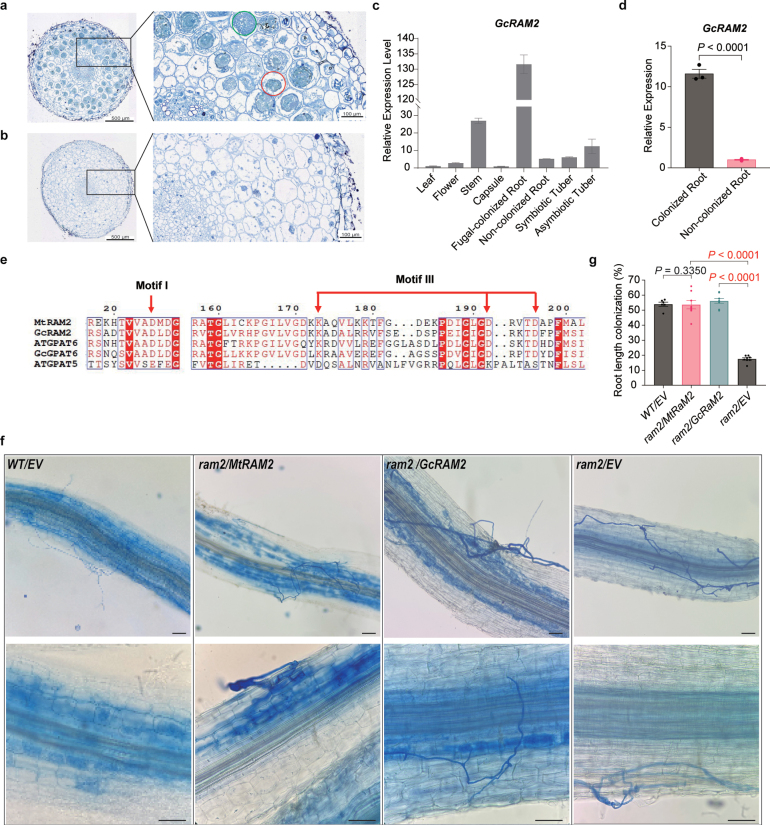
*GcRAM2* participates in *OrM* symbiosis, and its fatty acid biosynthesis function is conserved. **a, b** Longitudinal sections of roots collected from adult *G. conopsea* in natural habitats, stained with toluidine blue, were used to confirm fungal colonization. **a** Fungal-colonized root showing fungal colonization in cortical cells **b** non-colonized root devoid of fungal colonization. Active (green circle) and senescent pelotons (red circle) are visible in the root cortex cells **c** Tissue-specific expression of *GcRAM2* in adult plants. Values represent means ± SEM from three independent experiments **d** Quantification of *GcRAM2* transcript levels was performed in GS2-colonized and uncolonized roots of seedlings obtained from the symbiotic germination of *G. conopsea* seeds with strain GS2 for 5 months. Values represent means ± SEM from three independent experiments, with 10–15 roots per biological replicate. Two-tailed two-sample *t*-test. Significance was defined as *p* < 0.05 **e** Sequence alignment of the phosphatase domain of *RAM2*, *GPAT5* (lacking phosphatase activity), and *GPAT6* (active phosphatase). Motifs I and III are essential for phosphatase activity **f** Images of lactophenol trypan blue-stained arbuscules in *M. truncatula* roots of *ram2*/*EV* (A17), *ram2*/*MtRAM2*, and *ram2*/*GcRAM2* lines at 8 weeks post-inoculation (wpi) **g** Quantification of *R. irregularis* colonization in *WT/EV* (A17), *ram2*/*MtRAM2*, *ram2*/*GcRAM2*, and *ram2*/*EV* (A17) lines at 8 wpi. Values represent means ± SEM from 6–8 independent experiments. Two-tailed two-sample *t*-test. Significance was defined as *p* < 0.05. Scale bars: 500 μm (**b** left) and 100 μm (**b** right); 200 μm (**f**).

The tissue-specific expression analysis of *GcRAM2* in adult plants revealed that it is highly expressed in fungal-colonized roots (Fig. 5a), and the relative expression level was 25 times higher than in non-colonized roots (Fig. 5b, c). In seedlings colonized with GS2, the expression level in colonized roots was also significantly higher than in non-colonized roots (Fig. 5d). By contrast, the homolog of *GcRAM2*, *GcGPAT6*, was significantly expressed in non-colonized roots of seedlings (Suppl. material 1: fig. S7a) and highly expressed in the capsule, flower, and non-colonized roots of adult plants (Suppl. material 1: fig. S7b). *In situ* hybridization of protocorms showed that the expression of *GcRAM2* colocalized with fungus GS2, with relatively high expression in the GS2-colonized cells (Suppl. material 1: fig. S7c).

**Figure 6.**
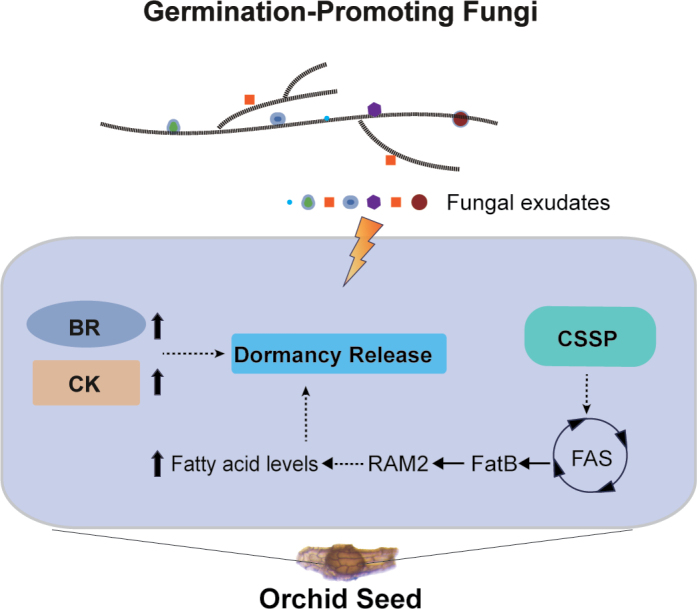
Fungal exudates elicit downstream transcriptional and physiological responses in *G. conopsea* seeds. Orchid germination-promoting fungi secrete a diverse array of complex molecules. Exposure to fungal exudates elevates phytohormone levels in seeds, including brassinosteroids (BR) and cytokinins (CK), as well as fatty acid levels, potentially facilitating orchid seed dormancy release. Orchids retain the core CSSP components shared with *AM*, and exposure to fungal exudates may increase fatty acid levels in *G. conopsea* seeds via this pathway.

*GcRAM2* is predicted to contain a functional phosphatase domain, similar to *MtRAM2* and *AtGPAT6*, which have been reported to be involved in cutin biosynthesis (Fig. 5e). *GcRAM2* and *MtRAM2* were overexpressed to test whether these transgenes could complement the *ram2* mutant. Following inoculation with *Rhizophagus irregularis*, the *ram2* mutant roots expressing *GcRAM2* exhibited high levels of colonization, which is consistent with the biological function of *MtRAM2* (Fig. 5f, g).

## Discussion

### *G. conopsea* seeds exhibit downstream physiological and transcriptional responses upon exposure to fungal exudates

Fungal exudates were found to influence the synthesis and signaling pathways of hormones potentially involved in seed germination, such as BR, CK, indole-3-acetic acid (IAA), and gibberellin (GA) (Suppl. material 1: figs S2e, S5), as well as metabolite and energy transformations, including starch and sucrose metabolism and fatty acid biosynthesis. The enrichment of genes associated with catalytic, oxidoreductase, and carbohydrate metabolic activities underscores the potential role of fungal exudates in metabolic reprogramming that drives seed dormancy release, which may explain the requirement for fungal assistance during *G. conopsea* seed germination.

### Endogenous phytohormones are potentially involved in orchid seed symbiotic germination

Seed germination is tightly regulated by diverse endogenous hormones, a mechanism likely conserved across seed plants (Miransari and Smith 2014; Penfield 2017). ABA and GA are key hormones regulating seed dormancy and germination, with ABA promoting dormancy and GA facilitating germination (Finkelstein et al. 2008; Ali et al. 2022). BR, CK, and auxins also play essential roles in cell division, elongation, and differentiation, supporting the transition from dormancy to active growth (Kucera et al. 2005; Ma et al. 2024).

A time-series transcriptomic analysis of the *G. conopsea*–GS2 symbiosis revealed that the early pre-symbiotic phase was characterized by significant phytohormone signal transduction activity (Zhao et al. 2025a), likely triggered by fungal-derived molecules before physical contact (Suppl. material 1: fig. S2c, d). The results demonstrated that exposure of seeds to symbiotic fungal exudates triggers plant hormone signal transduction and elevates BR and CK levels. Furthermore, exogenous BR and CK treatments significantly enhanced the symbiotic germination rates of *G. conopsea* seeds. In contrast, inhibition of BR biosynthesis in seeds drastically reduced fungal colonization, whereas suppression of CK biosynthesis led to a marked reduction in protocorm size. BRs enhance *AM* symbiosis by promoting root hair formation, fungal colonization, and nutrient exchange, while also activating the antioxidant system to mitigate oxidative stress (Ren et al. 2021; Ren et al. 2024). Similarly, CKs regulate nodule formation and *AM* symbiosis by promoting root hair development and cell division, while interacting with auxin, ethylene, and GA to coordinate symbiotic processes and stress responses during fungal colonization (Frugier et al. 2008; Anand et al. 2022). These findings suggest that both BR and CK likely positively regulate this process. However, the potential interactions between these hormones and the underlying regulatory mechanisms during orchid seed symbiotic germination remain to be fully elucidated, which will require the development of efficient fungal or plant genetic transformation systems.

Three bioactive GAs were detected in seeds treated with fungal exudates: GA_3_, GA_4_, and GA_7_. Notably, bioactive GA_7_ accounted for more than 99.9% of the total GAs, and its content increased significantly after treatment (Suppl. material 1: fig. S5a–c). Concurrently, GA transcriptional regulators were activated, while ABA signaling was repressed within the plant hormone signal transduction pathway (Suppl. material 1: fig. S2e). Although recent studies have suggested that exogenous GAs inhibit hyphal colonization in *AM* symbiosis and orchid seed germination (Chen et al. 2020; Floss et al. 2013), fungal colonization depends on DELLA proteins, which promote arbuscule development (Pimprikar et al. 2016). Elevated GA levels during the early stages of orchid seed germination suggest a role in breaking dormancy (Chen et al. 2020). As fungal colonization progresses, endogenous bioactive GA is converted into its inactive form, facilitating acceptance of the symbiotic partner (Miura et al. 2023).

The starch and sucrose metabolism pathway is enriched in *G. conopsea* seeds treated with fungal exudates (Fig. 2a), consistent with observations in *Gastrodia elata* seed germination (Yuan et al. 2025). Starch degradation in the endosperm, mediated by GA signaling, is critical for seed germination (de Lumen and Chow 1991; Finch-Savage and Leubner-Metzger 2006). Glucose, the primary product of starch degradation, attenuates ABA signaling (Faix et al. 2012), while its deficiency can induce GA signaling (Lu et al. 2007). Trehalose, glucose, and mannitol are abundant endogenous soluble carbohydrates in fungi (Patel and Williamson 2016). Orchid trehalase converts fungal trehalose into glucose, supporting germination (Zhao et al. 2024). The addition of exogenous sugars enhances orchid seed germination (Helmich et al. 2025). Although exudate-treated *G. conopsea* seeds enter the pre-germination stage, they do not fully germinate, possibly due to insufficient starch and free sugars or the need for a continuous influx of fungal metabolites. Further studies are required to determine whether orchid seeds have lost key metabolic genes and to elucidate the regulatory roles of hormones and carbohydrates in germination.

The results demonstrated that the IAA signaling pathway was significantly activated by fungal exudates (Suppl. material 1: fig. S2e) and that endogenous IAA was detected in *G. conopsea* seeds, but its content did not change significantly following treatment (Suppl. material 1: fig. S5d). Previous studies have confirmed that *Mycena* species supply IAA to the fully mycoheterotrophic orchid *Gastrodia elata*, thereby facilitating its seed germination (Yuan et al. 2025). However, the trophic strategies employed by fully and partially mycoheterotrophic orchids (e.g., *G. conopsea*) remain to be further validated. Furthermore, the role of IAA in orchid seed germination requires broader investigation.

The regulation of phytohormones warrants further investigation in orchid symbiosis. Specifically, which fungal signals induce hormone signaling during the pre-germination stage and how hormone signaling interacts with fungal colonization remain to be elucidated.

### Exudate exposure increases fatty acid levels in *G. conopsea* seeds potentially via the CSSP

Radhakrishnan et al. (2020) demonstrated, via analyses of 271 transcriptomes and 116 genomes, that *OrM* shares the core components of the CSSP with *AM.* Notably, these key genes are persistently expressed during orchid seed symbiotic germination, and the *CCaMK* gene can complement symbiosis-defective mutants in leguminous plants (Miura et al. 2018)—validating the conservation of this molecular mechanism. In orchid symbiosis, the continuous symbiotic trajectory encompassing seeds, protocorms, lateral roots, and tubers (Smith and Read 2008; Gao et al. 2023) aligns with the structural logic of *AM* roots (Gutjahr et al. 2008; Pumplin et al. 2010), in which meristems sustain fungal colonization via a continuous supply of new cortical cells. This provides a molecular evolutionary and morpho-developmental foundation for extending *AM* symbiosis theory to *OrM.*

CSSP genes in *G. conopsea* were transcriptionally induced prior to colonization by the GS2 fungus and remained highly expressed post-colonization (Fig. 4e), indicating that these genes play critical roles in the symbiotic germination of *G. conopsea* seeds. Furthermore, conserved symbiotic genes, including CSSP genes and *RAM2*, are evolutionarily conserved across multiple partially mycoheterotrophic orchids (*Apostasia shenzhenica*, *Vanilla planifolia*, and *Phalaenopsis equestris*) (Fig. 4f). These orchids retain the CSSP, which mediates successful colonization by orchid mycorrhizal fungi such as *Tulasnella*. This is consistent with previous reports that mycorrhizal symbiosis in partially mycoheterotrophic orchids depends on the CSSP (Miura et al. 2018).

Nevertheless, the CSSP is likely not essential for all *OrM* associations, as these genes are absent in fully mycoheterotrophic orchids (*Neottia fugongensis*, *Gastrodia elata*) (Fig. 4f, Suppl. material 1: fig. S6). Fully mycoheterotrophic orchids have evolved highly specialized mycorrhizal symbioses, e.g., the *Gastrodia elata*–*Mycena* symbiosis (Suetsugu et al. 2020), and may use a simpler signaling system instead of the CSSP.

Beyond the CSSP, a set of *AM*-specific genes also show targeted evolutionary differentiation in *OrM.* This pattern further demonstrates that *OrM* has selectively recruited and functionally adapted the conserved genes originally involved in *AM* symbiosis. *FatM*, *RAM1*, *STR*, and *STR2* are well-characterized *AM*-specific genes. Among them, *RAM1* is a key transcription factor that regulates *AM* symbiosis, *RAM2*/*FatM* participates in symbiosis-related lipid synthesis, and *STR*/*STR2* mediates lipid transport to *AM* fungi (Bravo et al. 2017; Jiang et al. 2017; Luginbuehl et al. 2017). All these genes have been lost in *OrM*, *EcM*, and *ErM* (Fig. 4f). They are specific to *AM* symbioses with *Glomeromycota* (Delaux et al. 2014) and unnecessary for orchids symbiotic with distantly related *Basidiomycota*/*Ascomycota*, representing adaptive evolution for orchid-specific mycorrhizae.

### Functional importance of RAM2 in lineages retaining colonization machinery

The symbiotic genes *FatM* and *RAM2* encode lipid biosynthetic enzymes that are essential for symbiosis, and these genes are uniquely conserved in plants that engage in *AM* symbiosis (Jiang et al. 2017; Jiang et al. 2018). *GcRAM2* is evolutionarily conserved and retains lipid synthesis function, directly confirming that the *AM*-derived *RAM2* gene maintains core functions after recruitment by *OrM.* Combined with the upregulated *RAM2* expression in highly mycoheterotrophic albino mutants of the partially mycoheterotrophic orchid *Epipactis helleborine* (Suetsugu et al. 2017), this reveals that *RAM2* remains functionally important in orchid lineages with conserved fungal colonization machinery. This is supported by its ability to complement the phenotype of the *ram2* mutant in *Medicago truncatula* (Fig. 5), a phenomenon also reported in *EcM* (Li et al. 2024). Given the widespread loss of *FatM* in *OrM*, functional replacement genes may exist. It was found that *GcFatB* and *GcRAM2* were similarly induced by exudates (Fig. 2b) and remained highly expressed during the symbiotic process (Wang et al. 2026), suggesting that *GcFatB* may act as a functional compensator for fatty acid synthesis after *FatM* loss, cooperating with *RAM2* to meet the lipid demands of *OrM* symbiosis—an evolutionary strategy for *OrM* to adapt to the absence of *AM* genes. Additionally, a shift in lipid accumulation within fungal tissues was observed, with the fungus putatively acquiring fatty acids from the non-photosynthetic orchid protocorms (Wang et al. 2026; Zhao et al. 2025a). Collectively, these findings indicate that *GcFatB* and *GcRAM2* are involved in fatty acid synthesis and may be related to fungal colonization of orchid seeds.

### LCOs might play a role in OrM associations

The symbiotic signals identified in fungal exudates so far are primarily carbohydrate-based molecules, such as chitin and lipopolysaccharides (Feng et al. 2019). LCOs released by rhizobia, *AMF*, or *EcM* fungi have been recognized as crucial signaling molecules that facilitate communication with host plants, enabling microbial partners to enter plant cells (Oldroyd and Downie 2004; Maillet et al. 2011; Cope et al. 2019). The root hair branching bioassay was used to validate that multiple fungi from *OrM*, including *Ceratobasidium* strains and *Tulasnella* strains (Suppl. material 1: fig. S3a), all exhibited the presence and activity of potential LCOs in their exudates.

Although the presence of LCOs has been proposed, their identification remains challenging due to their low natural abundance, complex chemical structures, and the need for highly sensitive detection methods (Bonhomme et al. 2021; Maillet et al. 2011; Tian et al. 2022), complicating their extraction, purification, and structural characterization. Overcoming these identification challenges and providing mass spectrometry data in the future will further confirm the presence of LCOs or other signal molecules, and such analyses are expected to verify which specific signals mediate the *OrM* symbiosis response.

### Future perspectives

Deciphering the orchid symbiotic machinery is constrained by the limited availability and efficiency of genetic transformation systems in orchids. Despite the successful application of *Agrobacterium*-mediated transformation to a few commercial orchid genera, including *Phalaenopsis* (Semiarti et al. 2007), *Cymbidium* (Chin et al. 2007), and *Dendrobium* (Teixeira da Silva et al. 2016), the overall transformation efficiency remains low. Recent studies (e.g., Subchan et al. 2022; Tiwari et al. 2024) confirm that enhancing transformation efficiency and streamlining culture protocols remain the central challenges in the genetic improvement of orchids. Future advancements are anticipated to overcome these limitations.

Orchid seeds rely on specific mycorrhizal fungi for carbon and nutrient acquisition during germination, resulting in low natural recruitment and germination rates. Based on the findings, elucidating exogenous hormonal regulation and characterizing symbiotic signals and their receptors will help unravel the molecular mechanisms underlying seed germination and colonization, enhancing *in situ* germination success via hormone or chemical treatments and advancing orchid ecology, reproductive biology, and conservation efforts. Integrative multi-omics approaches, including transcriptomics, metabolomics, and functional genetics, are anticipated to uncover conserved and lineage-specific modules of orchid–fungus crosstalk, thereby supporting orchid conservation and breeding strategies.

## Additional information

### Artificial Intelligence (AI) use

The authors accept full responsibility for the content of the manuscript, including the disclosure of any use of AI.

No AI tools were used in the preparation of this manuscript.

### Adherence to national and international regulations

All the fungal strains used in this study have been legally obtained, respecting the Convention on Biological Diversity (Rio Convention).

### Author ORCIDs

Y. Wang https://orcid.org/0009-0003-1202-072X

X. Qian https://orcid.org/0009-0003-4735-6362

J. Li https://orcid.org/0009-0005-9906-9589

H. Wang https://orcid.org/0009-0000-4551-3414

A. Yang https://orcid.org/0009-0002-1056-3571

L. Yang https://orcid.org/0009-0008-6523-9182

J. Liu https://orcid.org/0009-0001-1702-3474

X. Xing https://orcid.org/0000-0002-1845-5076

### Data availability

The raw transcriptome sequencing data of GS2 exudate-treated Gymnadenia conopsea seeds and Trinity de novo transcriptome data from this study were deposited in the National Genomics Data Center, China National Center for Bioinformation/Beijing Institute of Genomics, Chinese Academy of Sciences. Raw reads are accessible in the Genome Sequence Archive (GSA: CRA032664), and transcriptome assembly, expression and annotation data are available in the OMIX database (OMIX014458).

## Supplementary materials

Supplementary material 1 Supplementary figures Data type docx Explanation note **fig. S1**. A maximum-likelihood tree inferred from rDNA-ITS sequences of *Ceratobasidium* (**a**) and *Tulasnella* (**b**) strains. **fig. S2**. RNA-seq profiling of *G. conopsea* seeds. **fig. S3**. Root hair branching assays and untargeted metabolomic analysis of GS2 fungal exudates. **fig. S4**. Statistical analysis of protocorm length and width of *G. conopsea* under exogenous treatments with EBR, BRZ, 6-BA, and zeatin at 30 dps. **fig. S5**. Profiles of endogenous bioactive GAs (**a–c**) and IAA (**d**) in the control and exudate-treated groups of *G. conopsea* seeds. **fig. S6**. Evolutionary analysis using the maximum-likelihood method of CSSP genes across species with *AM*, *EcM*, *OrM*, *AM*+*EcM*, and *AM*+*RNS* symbioses. **fig. S7**. Expression localization of *GcRAM2* and tissue-specific expression of the homologous gene *GcGPAT6*. https://binary.pensoft.net/file/1718659This dataset is made available under the Open Database License (http://opendatacommons.org/licenses/odbl/1.0/). The Open Database License (ODbL) is a license agreement intended to allow users to freely share, modify, and use this Dataset while maintaining this same freedom for others, provided that the original source and author(s) are credited. Yaoyao Wang, Xin Qian, Jiaxin Li, Haoran Wang, Aiyiwei Yang, Luna Yang, Jiaxin Liu, Xiaoke Xing

Supplementary material 2 Supplementary tables Data type xlsx Explanation note **table S1**. Fungal internal transcribed spacer (ITS) sequences. **table S2**. Untargeted metabolomics analysis of GS2 exudates. **table S3**. KEGG pathway enrichment analysis for differentially expressed genes in the control vs. treatment group. **table S4**. Gene IDs and annotation information in transcriptome data. **table S5**. Time-series transcriptomic profiles from the symbiotic germination of *Gymnadenia conopsea* seeds. **table S6**. Identification of phytohormones in seeds treated with GS2 exudates. **table S7**. Primer sequences. https://binary.pensoft.net/file/1718660This dataset is made available under the Open Database License (http://opendatacommons.org/licenses/odbl/1.0/). The Open Database License (ODbL) is a license agreement intended to allow users to freely share, modify, and use this Dataset while maintaining this same freedom for others, provided that the original source and author(s) are credited. Yaoyao Wang, Xin Qian, Jiaxin Li, Haoran Wang, Aiyiwei Yang, Luna Yang, Jiaxin Liu, Xiaoke Xing
