# Supplementary material for: Ceratobasidium sp. GS2 exudates elicit downstream transcriptional and physiological responses in seeds of Gymnadenia conopsea (Orchidaceae)
Source: IMA Fungus. 2026 Jul 17;17:e191381. doi: 10.3897/imafungus.17.191381 (PMC13401186; doi:10.3897/imafungus.17.191381)
Supplement: Supplementary material 1 — Supplementary figures [file imafungus-17-e191381-s001.docx]

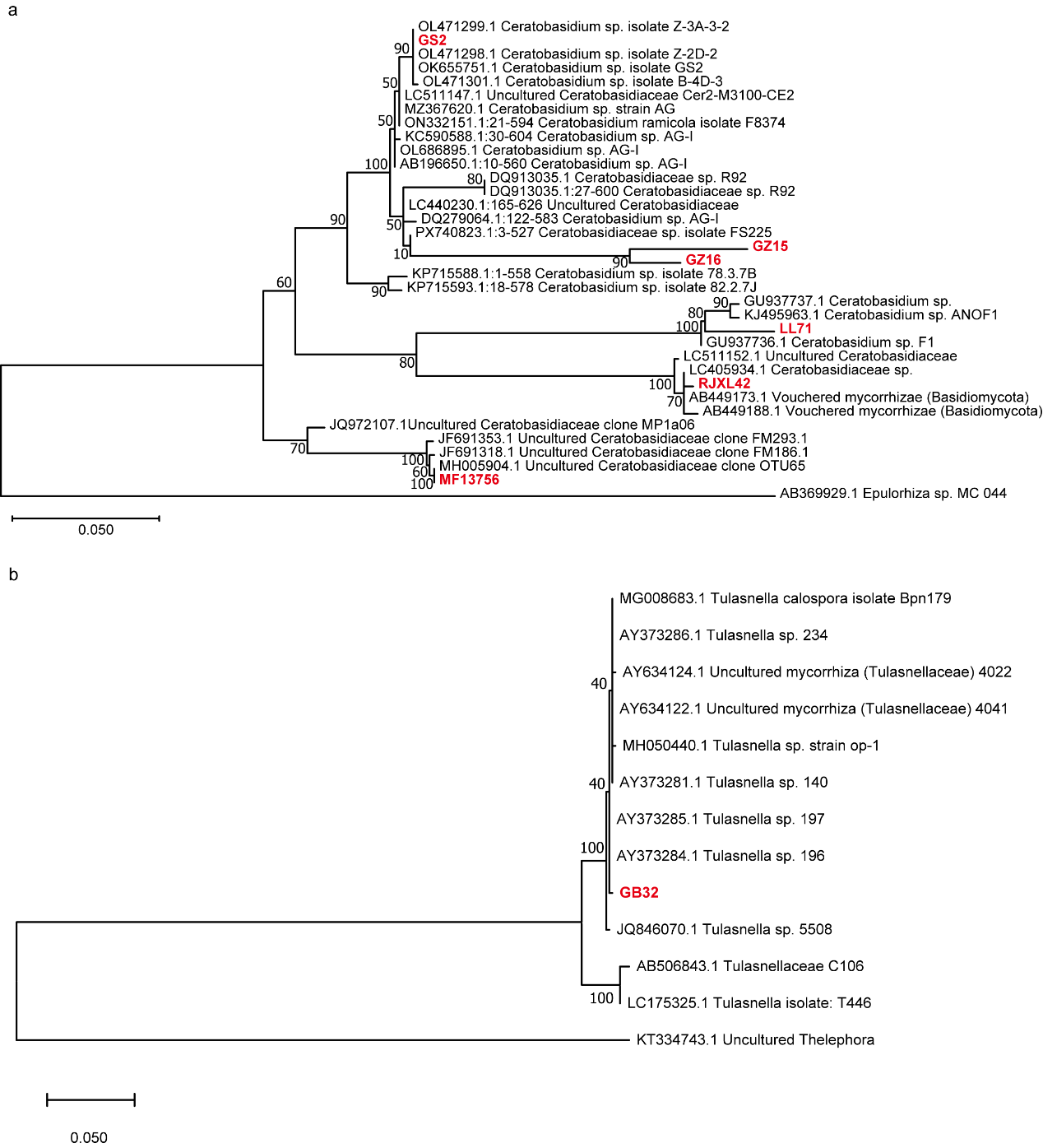


**Figure S1.** A maximum-likelihood tree inferred from rDNA-ITS sequences of *Ceratobasidium* (**a**) and *Tulasnella* (**b**) strains. Strains characterized in this study are highlighted in red.

**
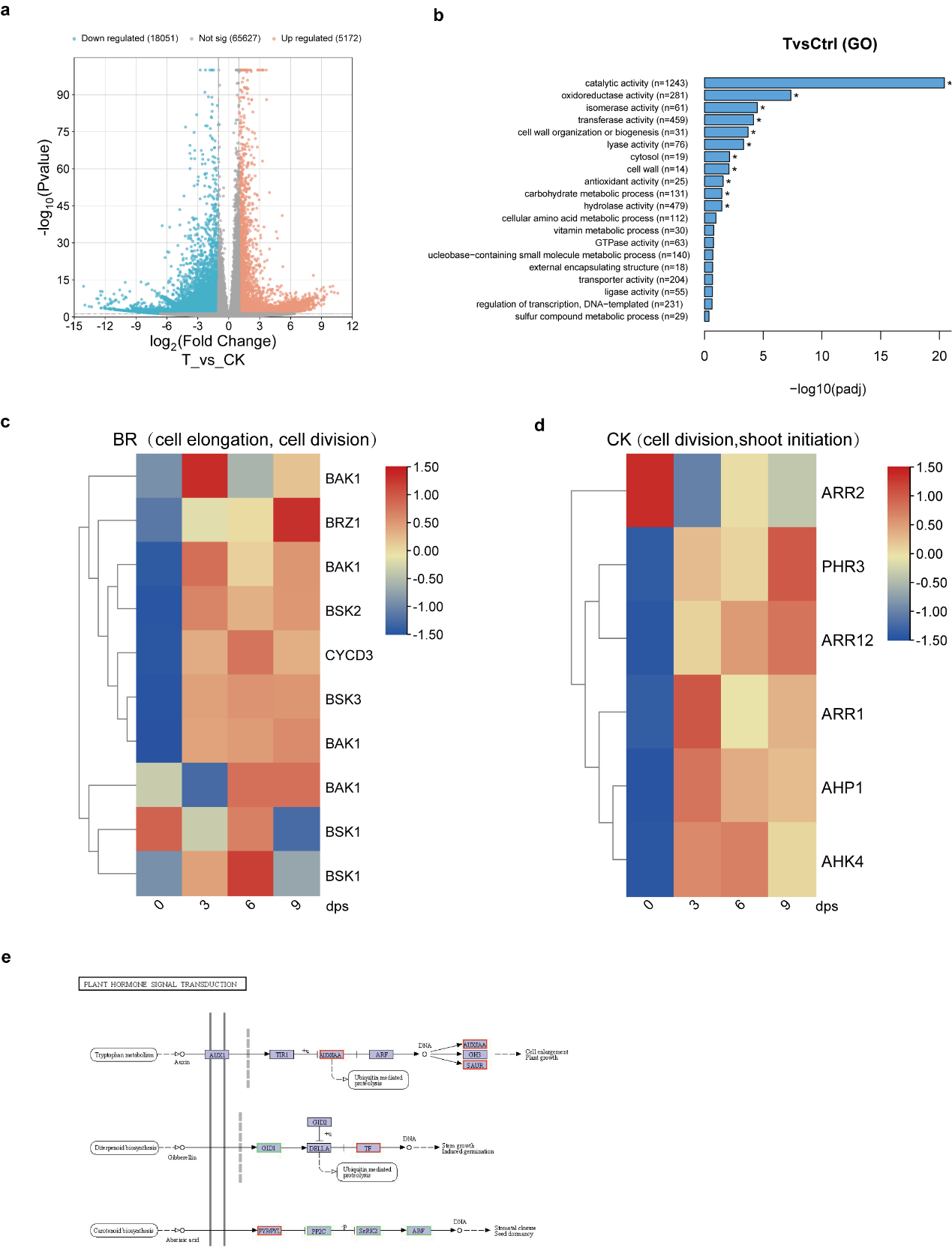
**

**Figure S2.** RNA-seq profiling of *G. conopsea* seeds. **a** Volcano plot of differentially expressed genes (DEGs) in *G. conopsea* seeds treated with GS2 exudates vs. CK (control) (|log₂FC| ≥ 1, adjusted *p* value ≤ 0.05). **b** Gene Ontology enrichment analysis of DEGs between treated (T) and CK samples. **c, d** Heatmaps showing the expression patterns of genes associated with the plant hormone signal transduction pathways of BR and CK signaling in symbiotic germination samples collected at 0, 3, 6, and 9 dps. **e** Plant hormone signal transduction (auxin, GA, and ABA) in *G. conopsea* seeds in response to fungal exudates.

**
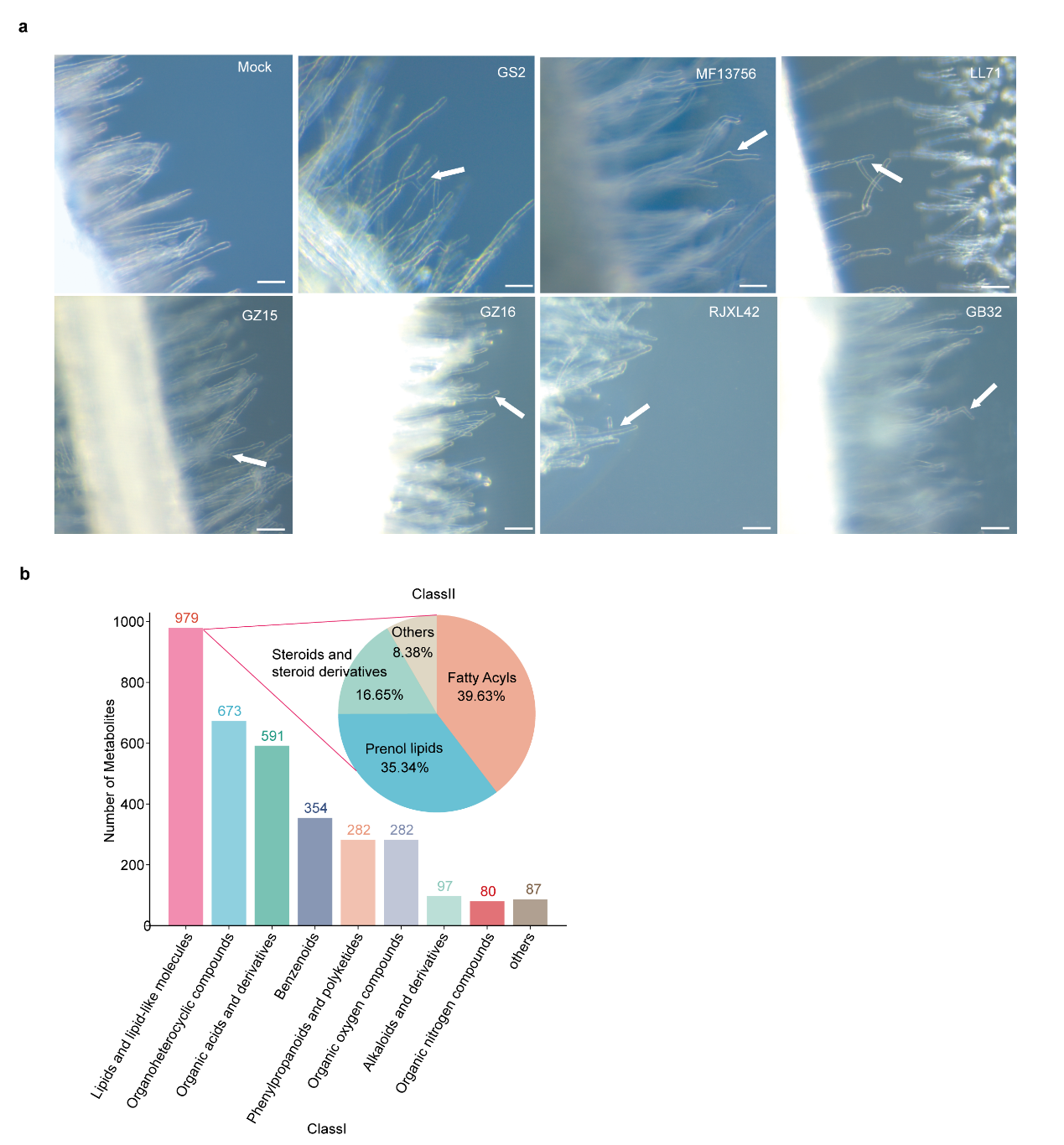
**

**Figure S3.** Root hair branching assays and untargeted metabolomic analysis of GS2 fungal exudates. **a** Representative images from root hair branching assays showing *M. truncatula* roots exposed to exudates from *Ceratobasidium* and *Tulasnella* strains of *OrM* fungi. Scale bar = 100 μm. **b** Classification of metabolites identified in GS2 exudates based on untargeted metabolomic analysis.


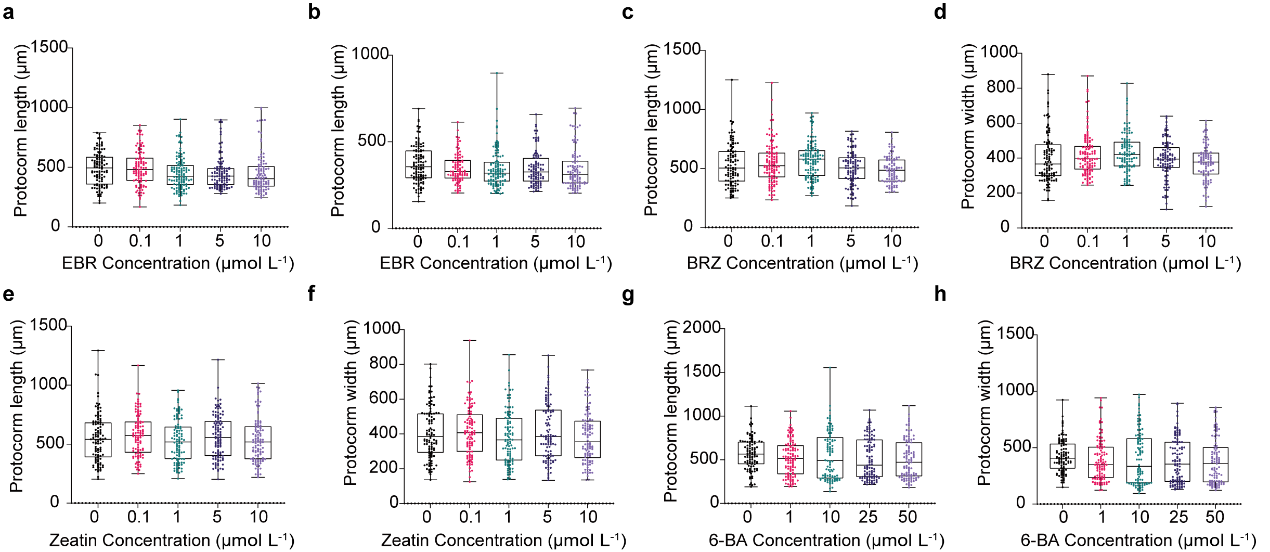


##### **Figure S4.** Statistical analysis of protocorm length and width of *G. conopsea* under exogenous treatments with EBR, BRZ, 6-BA, and zeatin at 30 dps. Bars represent mean ± SEM (*n* = 90–100 protocorms). Statistical significance was determined using one-way ANOVA followed by Tukey’s test. Significance was defined as *p* < 0.05.


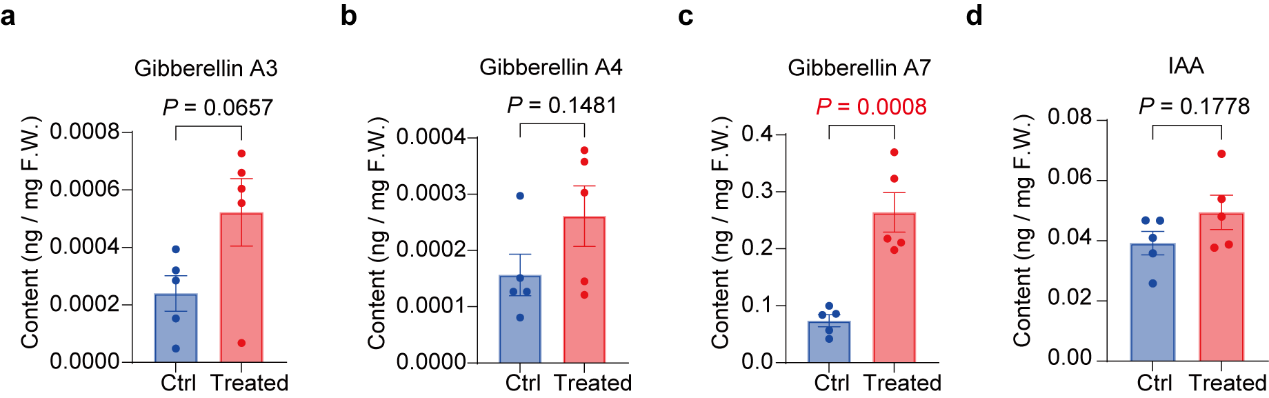


**Figure S****5.** Profiles of endogenous bioactive GAs (**a–c**) and IAA (**d**) in the control and exudate-treated groups of *G. conopsea* seeds. Bars represent mean ± SEM (*n* = 5). Two-tailed two-sample *t*-test. Significance was defined as *p* < 0.05. F.W., fresh weight.


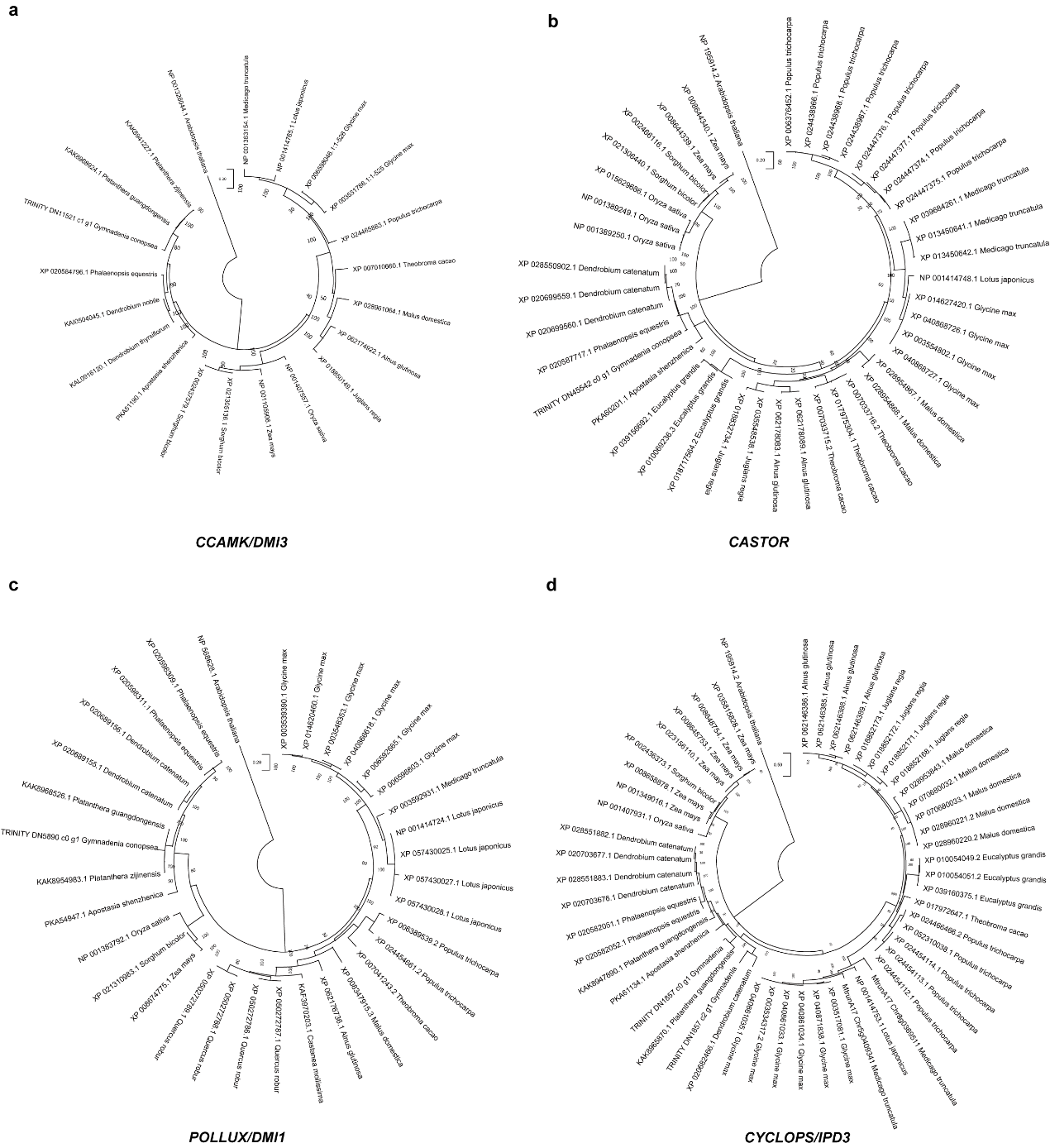


**Figure S6.** Evolutionary analysis using the maximum-likelihood method of CSSP genes across species with *AM*, *EcM*, *OrM*, *AM*+*EcM*, and *AM*+*RNS* symbioses. Maximum-likelihood trees show the phylogenetic relationships among the protein sequences of (**a**) *CCaMK/DMI3*, (**b**) *CASTOR*, (**c**) *POLLUX/DMI1*, and (**d**) *CYCLOPS/IPD3*. The accession numbers from the National Center for Biotechnology Information (NCBI) Reference Sequence are given for all sequences. Species included: *Medicago truncatula*, *Lotus japonicus*, *Glycine max*, *Oryza sativa*, *Sorghum bicolor*, *Zea mays*, *Theobroma cacao*, *Malus domestica*, *Alnus glutinosa*, *Populus trichocarpa*, *Castanea mollissima*, *Quercus robur*, *Juglans regia*, *Eucalyptus grandis*, *Apostasia shenzhenica*, *Dendrobium nobile*, *Dendrobium catenatum*, *Phalaenopsis equestris*, *Gymnadenia conopsea*, *Platanthera guangdongensis*, *Platanthera zijinensis*, and *Arabidopsis thaliana*.


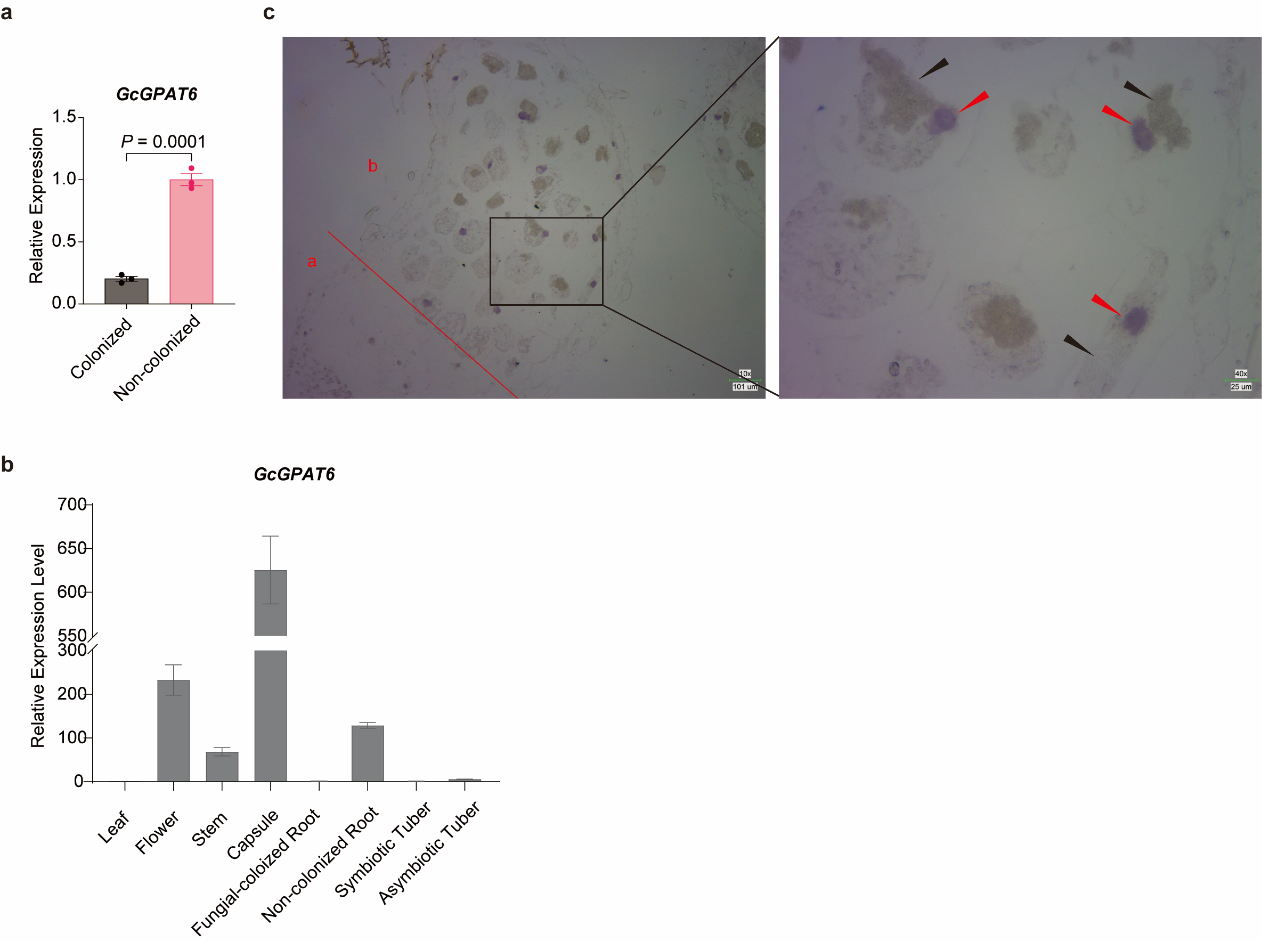


**Figure S7.** Expression localization of *GcRAM2* and tissue-specific expression of the homologous gene *GcGPAT6*. **a** Quantification of transcript levels of *GcGPAT6* in mycorrhizal and non-mycorrhizal roots of *G. conopsea* seedlings at 5 months post-symbiosis. Values are means ± SEM from three independent experiments, with 10–15 roots for each biological replicate. Two-tailed two-sample *t*-test. Significance was defined as *p* < 0.05. **b** Tissue-specific expression of *GcGPAT6* in adult plants. Values are means ± SEM from three independent experiments. **c** *In situ* hybridization of *GcRAM2* in the *G. conopsea* protocorm. “a” indicates the chalazal end, and “b” indicates the micropylar end. The red arrows indicate positive signals, and the black arrows indicate fungal pelotons.
